# Systemic and local inflammatory response after implantation of biomaterial in critical bone injuries

**DOI:** 10.1590/acb383823

**Published:** 2023-10-13

**Authors:** Patricia Brassolatti, Cynthia Aparecida de Castro, Hugo Leonardo dos Santos, Isabelle Taira Simões, Luciana Almeida-Lopes, Juliana Virginio da Silva, Fernanda Oliveira Duarte, Genoveva Lourdes Flores Luna, Wladimir Rafael Beck, Paulo Sergio Bossini, Fernanda de Freitas Anibal

**Affiliations:** 1 Universidade Federal de São Carlos – Postgraduate Program in Evolutionary Genetics and Molecular Biology – Department of Morphology and Pathology – São Carlos (SP) – Brazil.; 2 Universidade Federal de São Carlos – Department of Morphology and Pathology – São Carlos (SP) – Brazil.; 3 Institute of Research and Education in the Health Area – São Carlos (SP) – Brazil.; 4 Universidade de São Paulo – Institute of Physics of São Carlos – São Carlos (SP) – Brazil.; 5 Universidade Federal de São Carlos – Department of Physiological Sciences – São Carlos (SP) – Brazil.

**Keywords:** Biocompatible Materials, Skull, Bone Regeneration, Immunity

## Abstract

**Purpose:**

To evaluate inflammatory response in critical bone injuries after implantation of the biomaterial composed of hydroxyapatite (HA)/poly (lactic-coglycolic acid) (PLGA)/BLEED.

**Methods:**

Forty-eight male Wistar rats (280 ± 20 grams) were divided into two groups: control group (CG), in which the animals do not receive any type of treatment; and biomaterial group (BG), in which the animals received the HA/PLGA/BLEED scaffold. Critical bone injury was induced in the medial region of the skull calotte with the aid of a trephine drill 8 mm in diameter. The biomaterial was implanted in the form of 1.5-mm thick scaffolds. Serum and calotte were collected at one, three and seven days.

**Results:**

Biomaterial had a significant effect on the morphological structure of the bone, accelerating osteoblast activation within three days, without causing exacerbated systemic inflammation. In addition, quantitative real-time polymerase chain reaction (qRT-PCR) analysis showed that BG induced upregulation of osteogenic genes such as runt-related transcription factor 2, and stimulated genes of inflammatory pathways such as tumor necrosis factor-α, on the first day without overexpressing genes related to bone matrix degradation, such as tissue inhibitor of metalloproteinases-1 and matrix metalloproteinase-9.

**Conclusions:**

The HA/PLGA/BLEED^®^ association can be used as a bone graft to aid bone repair, as it is capable of modulating expression of important genes at this stage of the repair process.

## Introduction

The consolidation of critical bone injuries is still an emerging clinical problem, because extensive tissue losses resulting from tumor resections and/or extensive fractures generally have biological and physiological limitations that impair the adequate tissue repair. In general, the process of bone consolidation or regeneration includes distinct and overlapping steps divided into coagulation, inflammation, neovascularization, bone callus formation, mineralization, and remodeling. Therefore, it requires a perfect interaction between different cell types and their signaling pathways^1^.

Recently, immune response together with inflammation has gained notoriety, once it has been considered a crucial step for adequate cell recruitment that predicts the evolution of its subsequent phases. In addition, studies suggest that osteogenesis process is linked to an adequate activation of the immune response through the release of inflammatory mediators, so that an exacerbated pro-inflammatory stimulus, for example, can impair the synthesis of the extracellular matrix and its subsequent mineralization^2^-^5^ since the extracellular matrix (ECM) is a dynamic bioenvironment with mechanical and biochemical properties involved in cell adhesion, proliferation and responses to growth factors^6^.

Inflammation is a complex process in which a variety of cells, including B and T lymphocytes, myeloid cells, epithelial cells, fibroblasts, endothelial cells, muscle cells and adipocytes interact with each other through associated molecules such as matrix metalloproteinases (MMPs), cytokines, chemokines, and growth factors. Infectious stimuli, surgical intervention, injury due to mechanical, electrical, chemical, or thermal trauma can lead to a dysregulation of inflammation, leading to an excessive production of pro-inflammatory cytokines^7^.

Tumor necrosis factor-α (TNF-α), for example, can induce secondary inflammation and act as a chemotactic agent, recruiting cells to the site of injury and stimulating the differentiation of mesenchymal stem cells that aid in osteogenesis^8^. However, it can also inhibit osteoblast function through runt-related transcription factor-2 (Runx-2) degradation^9^. Interleukin 6 (IL-6), on the other hand, recruits inflammatory cells to the site of injury and promotes activation of specific receptors in osteoblasts^10^,^11^, but its exacerbated expression can result in increased osteoclastogenesis. Similarly, anti-inflammatory cytokines also play an important role in the process, as IL-4 is related to increased collagen synthesis, alkaline phosphatase expression and mineralization process, but it can decrease cell differentiation when unbalanced. It also occurs with IL-10, which is involved in chondrocyte proliferation and differentiation processes via bone morphogenetic proteins, directly influencing bone formation, the process of osteogenesis^12^,^13^.

Thus, inflammation is the first step and of paramount importance for the evolution of the repair process as a whole, because, when unbalanced, it can influence the formation of the extracellular matrix and lead to an increase in bone resorption from inadequate cell signaling. It should be noted that communication between inflammatory cells (polymorphonuclear leukocytes and cells of the monocyte-macrophage-osteoclast lineage) and cells related to bone healing (cells of the mesenchymal-osteoblast lineage and vascular lineage) is essential for biological processes to occur perfectly in each of its phases^14^.

As an aid to this meticulous biological process, treatment alternatives arise from tissue engineering knowledge, through the development of biomaterials capable of acting as bone substitutes and providing mechanical and/or biological stimuli for functional tissue restoration. These biomaterials can interact with biological systems to treat, augment or replace any tissue, organ or function in the body^15^. In addition, they can act as a tool and support to enable spatial and temporal control of the cell to guide bone regeneration in challenging healing environments^1^.

Among the most studied materials are hydroxyapatite (HA) and poly (lactic-coglycolic acid) (PLGA), which have demonstrated satisfactory effects as they possess specific properties of biocompatibility and biodegradability. Also, they effectively participate in promoting mechanical strength accompanied by an adequate degradability offered by PLGA, which favors tissue/biomaterial interaction and helps with cell adhesion and proliferation necessary for the formation of a new tissue. However, little is investigated about agents capable of assisting or participating in initial processes such as coagulation and inflammation. In this sense, BLEED, a plant origin polysaccharide with hemostatic properties, when incorporated into the initial structure of HA/PLGA, can add biological value, becoming a composite material with greater capacity for tissue participation and interaction^16^-^22^.

In fact, in a previous study by our research group, the treatment of calvaria with HA/PLGA/BLEED scaffolds induced specific morphological and biological responses, improving cell connection and bone regeneration at 15, 30, and 60 days. Furthermore, the biomaterial was more effective for bone regeneration than HA/PGLA without the addition of BLEED or in the absence of a scaffold. These findings highlight the need to understand the initial process of systemic inflammation in bone repair with the HA/PLGA/BLEED scaffold, since it showed good performance in healing at 15, 30, and 60 days^23^.

Thus, the aim of this study was to evaluate both local and systemic inflammatory phase of the bone repair process of critical bone injuries in the calvaria of rats after implantation of the biomaterial formed by HA/PLGA/BLEED.

## Methods

### Experimental animals

Forty-eight male Wistar rats (*Rattus norvegicus*: var. Albinus, Rodentia, Mamalia) were used at three months of age and had a mean body mass of 260 ± 20 grams. The animals from the Central Biotério of the Universidade Federal de São Carlos (UFSCar) were kept in the experimental room at the Physiological Sciences Department of the same institution, kept in collective polypropylene cages, in a hygienic environment with controlled temperature at 22°C, light-dark cycles from 12 h-12 h, and free access to commercial-type feed and water. Animal studies were carried out after approval by the Institutional Committee on Ethics in Animal Use of the UFSCar (CEUA 051/2014).

**Figure 1:**
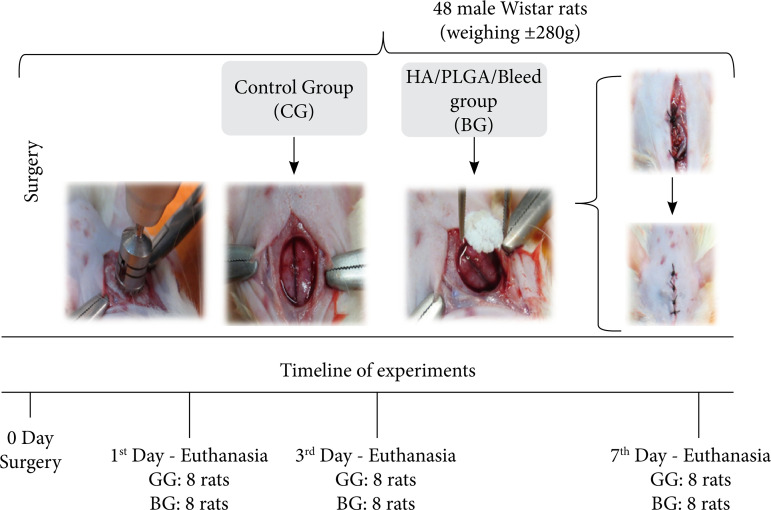
Experimental research design.

CG: control group; BG: biomaterial group HA: hydroxyapatite; PLGA: poly (lactic-coglycolic acid). Source: elaborated by the authors.

The animals were randomly distributed in two experimental groups with 24 animals each: control group (CG), in which the animals were induced to the bone defect of critical size and did not receive any type of treatment; and biomaterial group (BG), in which the animals were submitted to a bone defect and received the scaffolds implanted composed of the HA/PLGA/BLEED. Each experimental group was further divided into three subgroups, composed of eight animals each, with the intention of evaluating the repair process in different phases. Subgroup A was sacrificed on the first day after surgery, subgroup B was sacrificed on the third postoperative day, and subgroup C was sacrificed on the seventh postoperative day (Fig. 1).

### Preparation of composite HA/PLGA/BLEED

The biomaterial used in the study was developed with a HA/PLGA base. PLGA was dissolved in chloroform and placed in an ultrasonic bath. Then, the hydroxyapatite nanoparticles (induced by the calcium hydroxide treatment method), and Ca(OH)_2_ with orthophosphoric acid (H_3_PO_4_) were dispersed in this bath. Soon after, the mixture was placed on glass plates and allowed to evaporate in an oven at room temperature for 24 hours, being then transferred to a vacuum chamber, remaining there for 48 hours. For the formation of the new biomaterial, this HA/PLGA base was ground in a knife mill and sieved in an analytical granulometry sieve. Subsequently, the vegetable polysaccharide paste commercially known as BLEED (developed and manufactured exclusively by DMC Import and Export of Equipment LTDA) was added. At the end of the process, the suspension was freeze-dried to obtain scaffolds with 1.5 mm of thickness and 8 mm in diameter^23^.

### Sugical technique

For surgical procedures, the animals were weighed, previously shaved, and anesthetized with a combination of ketamine (95 mg/kg) and xylazine (12 mg/kg). After this process, an incision of approximately 1.5 cm was made in the medial region of the skullcap, in the anteroposterior direction. For the induction of a critical bone defect, a trifoil dental drill (WMA – Brazil) with 2 cm in length, 8 mm in external diameter and driven by a BELTEC micromotor (Araraquara, SP, Brazil) with a rotation of 13,500 rpm was used. The drill was positioned perpendicularly to the bone surface in order to break the external and internal cortices until the dura mater was exposed, creating a hole with a diameter of 8 mm. This process was set up under abundant saline irrigation. The scaffolds were placed in the created defect, immediately after the same, filling the defect completely. After the procedure, the suture was performed, and the animals received proper postoperative care^24^.

### Eutanasia of animals and collection of samples

Euthanasia was performed by decapitation, and the blood was separated at the first, third and seventh postoperative day, according to each experimental subgroup. The blood was centrifuged at 1,500 *g* for 15 min to separated the serum, and aliquots of the serum were frozen at -80°C for subsequent analyses. Immediately thereafter, the region of the critical-size bone defects area was removed and sent to the processing slides for further analysis.

### Histopathological analysis

For histopathological analysis of critical bone defects area, the extract material was fixed in 10% buffered formalin (Merck, Darmstadt, Germany) for 24 hours, decalcified in 4% EDTA solution and later embedded in paraffin. Sections were obtained in longitudinal orientation, with a thickness of 5 μm and mounted on histological slides. Slides were stained with hematoxylin and eosin (HE) (Merck, Darmstadt, Germany) for qualitative analysis of the bone defect area. The analyses were performed by a pathologist experienced in the specific area using a light microscope (Olympus, Optical Co. Ltd, Tokyo, Japan), and inflammatory changes, vascular changes, connective tissue, and bone matrix were observed.

### Cytokine measurement

The quantification of cytokines in serum was performed through the immunoenzymatic assay (enzime-linked immunosorbent assay–ELISA) by specific detection kits from BD OptEIA (BD Biosciences, Pharmingen, San Diego, United States of America), according to the manufacturer’s instructions. Briefly, the process occured by sensitization of 96-well plates, followed by the capture of the cytokines in the sample by the primary antibody present on the plate. Subsequently, a secondary antibody was used conjugated with peroxidase enzyme, to bind to these samples taken by the primary. After this used 3.3’, 5.5’ tetramethylbenzidine (TMB) substrate reacted with the enzyme conjugated to the secondary antibody by emitting the colorimetric signal. The reaction was blocked with the application of 50 μL/well of 2N sulfuric acid, and the absorbance was read in a wavelength of 450 nm in a microplate reader (Thermo Scientific: multiskan GO microplate spectrophotometer). The concentrations were calculated from a curve of the cytokine patterns, and the final concentrations were expressed in pg/mL or ng/mL.

### RNA isolation and cDNA synthesis

For the RNA isolation, calvaria were dissected, rapidly frozen in liquid nitrogen and stored (-80°C) until analysis. Total RNA was extracted from the bone defect using the Trizol reagent (1 mL, Invitrogen, Carlsbad, CA, United States of America) according to the manufacturer’s instructions. Total RNA was extracted from area of the critical-sized defect using Trizol reagent (Invitrogen, Carlsbad, CA, United States of America) according to the manufacture’s instruction. Potential DNA contamination was removed by DNase I (Cellco), and the purity was assessed by determining the ratio of the absorbance at 260 and 280 nm (MultiSkan Go with μDrop Plate Thermo Fischer). The total RNA (2 μg) was applied as template for cDNA synthesis using the high-capacity cDNA reverse transcription (Life Technologies, Carlsbad, CA, United States of America) following the manufacturer’s instructions. All real-time primers were initially tested against standards, and a standard curve was generated. Oligonucleotide primers were designed for ribosomal protein S18 (RPS18), TNF-α, matrix metalloproteinase-9 (MMP-9), tissue inhibitor of metalloproteinases-1 (TIMP-1), and Runx-2 (Table 1).

**Table 1:** Polymerase chain reaction primers used in this paper.

Gene	Sense	Antisense	Specifications
RPS18	ACTGCCATTAAGGGTGTG	GTCAGGGATCTTGTATTGTC	Sigma-Aldrich (NM_213557.1)
TNF-α	CACCACGCTCTTCTGTCTAC	ATCTGAGTGTGAGGGTCTGG	Sigma Aldrich (NM_012675.2)
MMP-9	TACTTTGGAAACGCAAATGG	GTGTAGAGATTCTCACTGGG	Sigma Aldrich (NM_031055.1)
TIMP-1	ATAGTGCTGGCTGTGGGGTG	TGATGCCTCTGGTAGCCCTT	Bioneer (NM_053819.1)
Runx2	AGAGTCAGATTACAGATCCC	TGTCATCATCTGAAATACGC	Sigma Aldrich (NM_001278483.1)

RPS18: ribosomal protein S18; TNF-α: tumor necrosis factor-α; MMP-9: matrix metallopeptidase 9; TIMP-1: metallopeptidase inhibitor 1; Runx2: runt-related transcription factor 2. Source: elaborated by the authors.

### Quantitative real-time polymerase chain reaction

The cDNA samples were subjected to quantitative real-time polymerase chain reaction (qRT-PCR) using an Aria Real-Time PCR System (Agilent) using Brilliant III Ultra-Fast qPCR Master Mix (Agilent). The optimized PCR conditions suffered initial denaturation at 95°C – 3 min (Hot Start) e 95°C – 5 s/60°C – 10 s (amplification) with 40 cycles de amplification. Negative control reactions with no template (deionized water) were also included in each run. For each gene, all samples were simultaneously amplified in duplicate in one assay run. Analysis of relative gene expression was performed using the 2^-∆∆ct^ method^25^. RPS18 was used as a housekeeping gene to normalize our expression data.

### Statistical analysis

The data obtained in this study were presented through descriptive analysis. The distribution of variables was tested using the normality (Kolmogorov-Smirnov method) and equal variance (Levene method). For the analysis of multiple comparisons, two-way analysis of variance (ANOVA) with Tukey’s post hoc tests were used to evaluate the variance between groups for parametric data (results were presented in mean and standard deviation) and nonparametric data, and the Kruskal-Wallis’ test was used with post hoc Dunn (results were presented as the median with the upper and lower quartiles: Me [Q1; Q3]). The relation between genes in the bone tissue was evaluated by linear regression and Pearson’s correlation analysis. Differences between the groups were considered significant when they obeyed a p ≤ 0.05 result. The statistical program used for all analyses was the Sigmaplot software (version 14).

## Results

### Histopathological analysis

**Figure 2:**
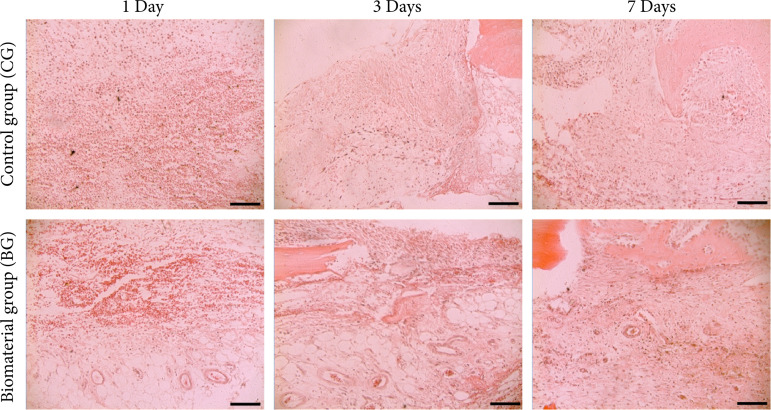
Representative histological sections of bone defect of critical size of the control and biomaterial PLGA/BLEED group at one, three, and seven days after surgery. Hematoxylin and eosin staining, 20x.

Source: Elaborated by the authors.

The results obtained by the histological analysis showed significant and interesting changes and differences among the groups. In general, in the three aspects evaluated, inflammatory infiltrate, vascular changes, and connective tissue and bone matrix, it was possible to observe that the recruitment and biological signaling verified in the BG presented a certain advance when compared to the CG. The detailed description of the results is presented in Table 2, and the representative photomicrographs are in Fig. 2.

**Table 2:** Histological description of inflammatory, vascular, and connective tissue/bone matrix alterations. Control and biomaterial PLGA/BLEED group at one, three, and seven days after surgery.

	Control group				Biomaterial group		
	Inflammatory alterations	Vascular alterations	Connective tissue/bone matrix		Inflammatory alterations	Vascular alterations	Connective tissue/bone matrix
1^st^ day	Activated macrophages, moderate eosinophilia, and free hemosiderin	Moderately focally extensive hemorrhage in lesion area	Moderate thickening of connective tissue associated with hemorrhage		Presence of macrophage with hemosiderin, eosinophils, plasmocytes, lymphocyte, discrete to moderate degree	Moderate to severe hemorrhage and congestion in lesion area, connective tissue dura mater, and arachnoid	Moderate to severe thickening of connective tissue and arachnoid associated with hemorrhage
3^rd^ day	Discrete and diffuse lymphoplasmacytic	Moderately focally extensive hemorrhage in lesion area, connective tissue and moderately entering the bone tissue	Moderately reactive dense connective (with neutrophils and macrophages)		Lymphocytes, plasmocytes and macrophages infiltrate, discrete to moderate in the connective tissue	Moderate hemorrhage located in the center of the lesion; diffuse hemorrhage in connective tissue, dura mater, and periosteum	Discrete necrosis in the center of the lesion, osteoblastic activity in the dura mater, thickening of connective tissue associated with hemorrhage
7^th^ day	Discrete and moderate lymphoplasmacytic	Moderately focally extensive hemorrhage in lesion area	Central bone cleft with *debri*; connective tissue and bone matrix thickening		Lymphocytes and plasmocytes infiltrate, discrete to moderate in the connective tissue and with a discrete degree close to the periosteum	Discrete to moderate and diffuse hemorrhage in connective tissue and pia mater	New bone formation (osteoblastic activity in the periosteum region), thickening of connective tissue mediated by lymphoplasmacytic infiltrate fibroblast activity

Source: Elaborated by the authors.

We observed that after three days the BG already showed osteoblastic activity, differently from the CG. In addition, on the seventh day we also verified a new bone formation with osteoblastic activity in regions close to the periosteum with connective tissue thickening and fibroblast activity. These are important features in bone formation and biomaterial incorporation. Another important factor was that, one day after the surgery, the BG showed a greater recoil of cells such as eosinophils, plasma cells, and lymphocytes.

### Cytokine measurement

It was noticed that, throughout the observed period, the CG presented greater variability compared to the BG, reaching greater amplitudes and being more heterogeneous for both IL-6 and TNF-α. In relation to the BG, the results were more homogeneous with little variability between the observed days, occurring practically in the same absorbance range. However, it is verified that on the first and seventh day there was reduction in the values of IL-6 and TNF-α, showing that it was the group with the lowest level of expression when compared to the CG.

**Figure 3:**
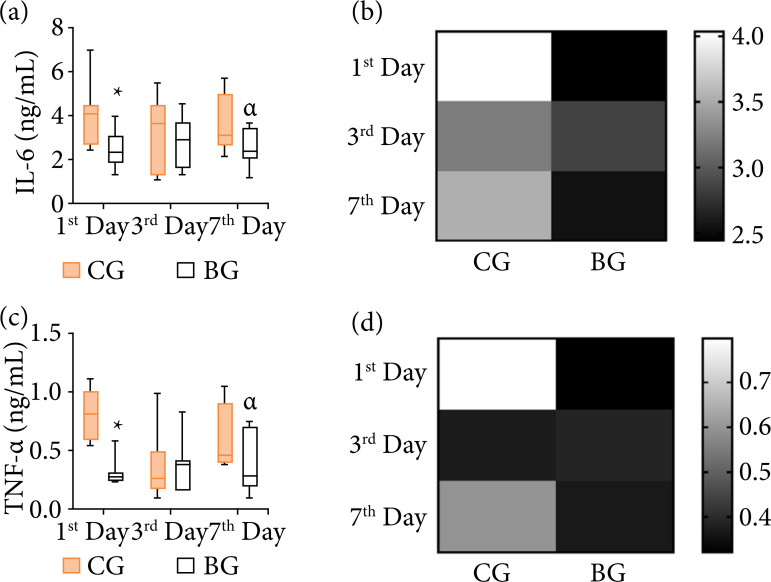
Serum pro-inflammatory cytokines in rats undergoing implantation of the scaffolds in different experimental periods. **(a)** IL-6 in ng/mL (Me [Q1; Q3]); **(b)** representation of group means to IL-6; **(c)** TNF-α in ng/mL (Me [Q1; Q3]); **(d)** representation of group means to TNF-α. P > 0.05.

IL-6: interleukin 6; TNF-α: tumor necrosis factor-α; CG: control group; BG: composite biomaterial group; *BG *versus* CG on the first day; αBG *versus* CG on the seventh day. Source: elaborated by the authors.

It is noteworthy that through the two-way ANOVA, for IL-6, we observed that there was a significant effect for the biomaterial factor (*F*:13.03; p = 0.001), but not for the time factor or even the interaction (biomaterial × time) (*F*:1.71; *p* = 0.188). This fact indicates that the difference in mean values between the different levels of treatment is greater than it would be expected by chance, after considering the effects of differences in time. That is, the biomaterial was able to reduce the concentration of systemic IL-6, and post hoc tests showed differences between groups at the first and seventh day after the implantation of the biomaterial (Fig. 3a).

The behavior of the means in the BG (Figs. 3b and 3d) when compared to the CG suggested a modulation of inflammation by the biomaterial, as it indicates that the biomaterial did not intensify the inflammation process. That is, it can be assumed that the biomaterial was not recognized by the organism as a foreign body, and with that its behavior can be considered biocompatible with the evaluated phase.

**Figure 4:**
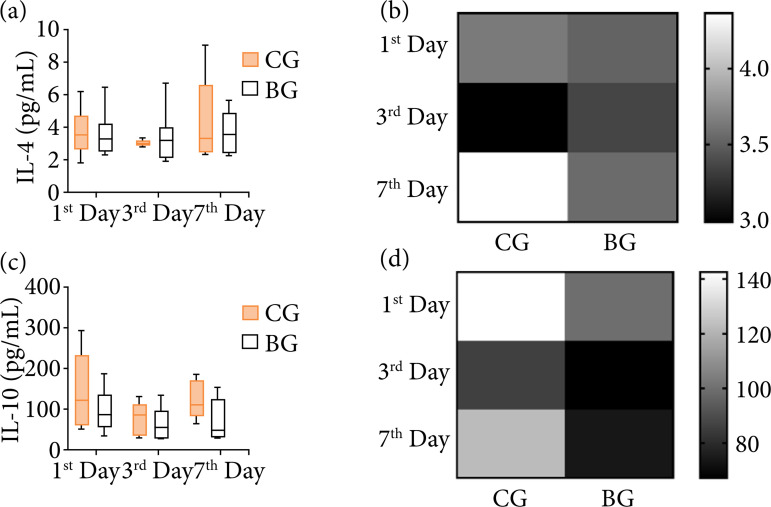
Serum anti-inflammatory cytokines in rats undergoing implantation of the scaffolds in different experimental periods. **(a)** IL-4 in pg/mL (Me [Q1; Q3]); **(b)** representation of group means to IL-4; **(c)** IL-10 in pg/mL (Me [Q1; Q3]); **(d)** representation of group means to IL-10. P > 0.05.

IL-4: interleukin 4; IL-10: interleukin 10; CG: control group; BG: composite biomaterial (HA/PLGA/BLEED); *BG *versus* CG on the first day; αBG *versus* CG on the seventh day. Source: elaborated by the authors.

Systemic anti-inflammatory response was evaluated with IL-4 and IL-10 (Fig. 4). It was noticed that, throughout the period observed, both groups showed great variability, reaching greater amplitudes and being more heterogeneous, especially for IL-4 (Figs. 4a). Thus, no statistical difference was found in the behavior of Il-4 and IL-10 (Figs 4a and 4c). Considering the behavior of the means in the BG (Figs. 4b and 4d) compared to the CG, it suggested a modulation of inflammation by the biomaterial, as that the biomaterial did not intensify the inflammation process.

### qRT-PCR evaluation

**Figure 5:**
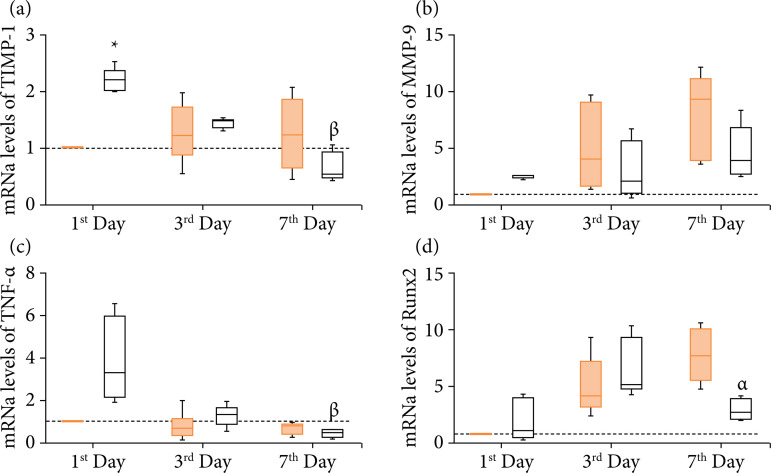
Gene expression via quantitative real-time polymerase chain reaction of **(a)** TIMP-1, **(b)** MMP-9, **(c)** TNF-α and **(d)** Runx2 genes in relation to the calibrator group (control 1 day). The data represent the mean ± standard deviation. p > 0.05.

TIMP-1: tissue inhibitor of metalloproteinases-1; MMP-9: matrix metalloproteinase-9; TNF-α: tumor necrosis factor-α; Runx2: runt-related transcription factor-2; CG: control group; BG: composite biomaterial (HA/PLGA/BLEED); *BG *versus* CG on the first day; αBG *versus* CG on the seventh day; βBG on the seventh day *versus* BG on the first day. Source: elaborated by the authors.

Figure 5 demonstrates gene expression for CG and BG after one, three, and seven days of implantation. One day after implantation, no difference was observed in the expression of the MMP-9, Runx-2 and TNF-α genes compared to BG with CG. However, in the same period, the TIMP-1 expression had a significant increase in the BG group compared to CG (Fig. 5a). Three days post-surgery, no difference in the expression of TIMP-1, MMP-9, Runx-2, and TNF-α was observed when comparing BG and CG (Figs. 5c and 5d). After seven days of implantation, no difference was observed in the gene expression of the TIMP-1, MMP-9, and TNF-α compared to BG with CG. However, in this period, BG presented a significant decrease in Runx2 expression when compared with CG.

**Figure 6:**
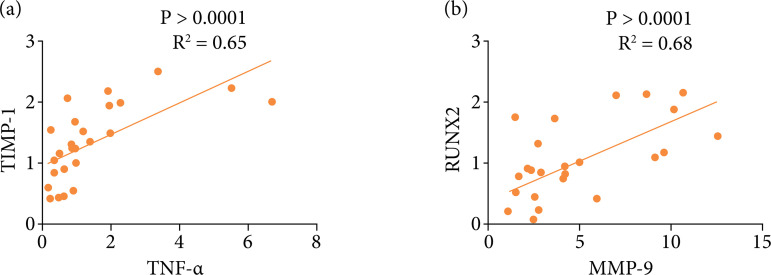
Graphic representation of the Pearson correlation between the expression ratio of **(a)** TNF-α/TIMP1 and**(b)** Runx2/MMP-9.

TIMP-1: tissue inhibitor of metalloproteinases-1; TNF-α: tumor necrosis factor-α; Runx2: runt-related transcription factor-2; MMP-9: matrix metalloproteinase-9. Source: elaborated by the authors.

It is worth mentioning that an expression of TIMP-1 and TNF-α in the BG decreased on the seventh day compared to BG on day 1. Besides that, we observed a moderate correlation between TIMP-1 values with TNF-α (Fig. 6a) and Runx2 with MMP-9 (Fig. 6b).

## Discussion

This study aimed to evaluate the initial stages of the repair process in critical bone defects, correlating local and systemic inflammation with bone repair genes in the first days after implantation of the new biomaterial HA/PLGA/BLEED. The main findings were that the use of biomaterial HA/PLGA/BLEED showed osteoblastic activity with only three days, unlike the CG. Furthermore, on the seventh day we observed the beginning of new bone formation based on osteoblastic activity in regions close to the periosteum with connective tissue thickening. As a complement, the qRT-PCR analysis showed that the biomaterial HA/PLGA/BLEED induced an upregulation of the extracellular matrix via stimulation of TIMP-1 on the first day, without unbalancing the expression of MMP-9, also stimulating a downregulation of Runx-2 and TNF-α on day 7. This scenario was accompanied by an equilibrium in the concentration of inflammatory cytokines during the time analyzed, which suggests that the biomaterial did not promote an unwanted or exacerbated immune response.

Such results indicate that inflammation was mild in the groups treated with the biomaterial in the initial periods when compared to the control. The study of the inflammatory behavior in the initial periods of the implantation of the biomaterial is of great relevance for the understanding of the interaction between biomaterial and tissue, which effectively predicts its biocompatibility, leading to adequate bone repair. Additionally, systemic inflammation was evaluated to understand whether there were inadequate immune responses when the organism came into direct contact with the implanted biomaterial. Biocompatibility is one of the main properties evaluated in this regard, since the host’s immune system is the first to respond to an antigen, which triggers a specific and complex immune response that is still poorly explored. The function and regulation of some cytokines occurs through the activation of biological cascades and it is directly related to growth factors essential for the proliferative phase, initiated mainly after the activation of an inflammatory response. High concentrations of IL-4 can inhibit bone remodeling^26^. On the other hand, the non-exacerbated release of IL-4 and IL-10, as occurred in our study, can suppress the immune response, evolving to a more adequate regeneration process^12^,^27^.

In turn, TNF-α and IL-6 are involved in the expression of the RANK-L ligand. This ligand is directly related to the bone resorption factor referred to and triggered by osteoclasts. Metabolically, bone is active throughout its life. Therefore, a strong balance between bone production and resorption factors is necessary, that is, between the synthesis of new matrix through the action of osteoblasts and its adequate resorption dependent on the activity of osteoclasts. With this, in the initial periods, any derangement arising from, for example, inflammatory cytokine cascades such as TNF-α and IL-6 is not advantageous, as these can delay the increase in the RANK-L factor and consequently increase the activity of the osteoclasts^26^,^27^. In-vitro studies proved that IL-6 promotes the genesis of osteoclasts, increasing the expression of RANK-L. High levels of IL-6 also promote the development of osteoprotegerin related to the control of bone metabolism, and such imbalance impairs bone resorption can trigger cellular apoptosis mechanisms. On the other hand, low levels of IL-6 are related to lower bone protection with signs of osteolysis^11^.

The biological mechanisms that control the balance of inflammatory cytokines are not yet fully understood, but cytokines seem to play an important role, and one of the main ones is TNF-α. The literature points out that TNF-α can potentially induce osteoclast formation by directly inducing osteoclast formation or stimulating the expression of RANK-L^28^. With this, there would be more bone resorption when compared to deposition, which would not be advantageous for the repair process. Thus, the reduction of TNF-α can be an important point to be scored, as was observed in our study both in the evaluation of the systemic concentration and in the transcription process of the local tissue after one day of the lesion establishment.

Thus, it is worth mentioning that the analysis by qRT-PCR revealed a positive regulation for TNF-α on the first day after the implantation of the biomaterial, but with reduced progression over the days, in which the BG on the seventh day showed lower levels of TNF-α than the BG on the first day after injury. Furthermore, histological analysis showed that this increase in TNF-α on the first day was necessary to stimulate the retreat of cells that participate in the initial repair process, such as macrophages, eosinophils, plasma cells and lymphocytes.

Similarly, studies indicate that the stimulation of TNF-α on the first day may be beneficial, as it initiates the recruitment of mesenchymal cells from the surrounding soft tissue, bone cortex, bone marrow, periosteum and blood cells with osteogenic and chondrogenic potential, but if TNF-α levels are not decreased after the second/third day there may be a diffuse impairment of chondrocyte proliferation and increased rates of chondrocyte apoptosis^2^,^26^,^27^. Another study also reinforces that in high concentrations it can lead to the inhibitory process^8^. Studies show that, in a murine model, the addition of TNF-α at the fracture site accelerates bone repair^8^, however, within 24 hours of the establishment of the lesion^29^, which may corroborate what was observed in our study, since the application of the biomaterial increased the levels of TNF-α at the lesion site.

Another relevant correlation is that TNF can inhibit osteoblast function through Runx-2 degradation^9^. In the present study, the expression values of Runx-2 were not altered after 24 hours of biomaterial implantation. On the contrary, there were a stimulus of increase in expression until the third day and then a reduction on the seventh day after implantation with the biomaterial. Such findings corroborate the study by Kido et al.^30^, who also observed a reduction in Runx-2 gene expression on the seventh day, using a PLGA biomaterial associated with biosilicate, and indicated that the stimulus to this factor returned on the 14^th^ day, which prevented any kind of deleterious effect on bone remodeling.

The histological analysis showed that seven days after implantation of the biomaterial tested in the present study the tissue already presented some characteristics that are correlated with good formation of the bone matrix, calcium deposition coming from an adequate granulation tissue, demonstrating an evolution of the inflammatory to proliferative. Such characteristics can be correlated with the expression values of Runx-2, that were not changed after 24 hours, which is important because osteoblast differentiation can be reduced by the overexpression of Runx2 through an increase in the expression of MMPs, such as MMP-9^31^, which also showed no changes. Interestingly, Runx-2 and MMP-9 are functionally linked^32^, so that Runx-2 has a key role in the transcriptional regulation of MMP-9 and that modulation of MMP-9 expression by Runx-2 influences cell migration^33^.

In turn, MMP-9 plays a crucial role in regulating chondrogenic and osteogenic cell differentiation during the early stages of bone repair. Elevated levels of MMP-9 indicate the beginning of the bone remodeling process and are maintained by the presence of osteoclasts that promote resorption and remodeling, facilitating the integration between tissue/graft^34^ or tissue/biomaterial. In this context, TIMPs are important for the balance between synthesis and degradation of ECM. Specifically, TIMP-1 is an endogenous inhibitor of bone matrix degradation that binds to active MMP-9, negatively regulating MMP-9 activity, thus controlling this process and also proteolysis^35^.

The present study demonstrated that the biomaterial stimulated a high expression of TIMP-1 in the first 24 hours, differently from the result found in the CG. Bone remodeling and repair comprise an extraordinarily complex sequential mechanism that initially requires the dynamic and intense breakdown of ECM components and directs cells involved in adhesion, controlling this mechanism and its regulation by proliferation and differentiation, which are decisive for the bone repair process mineralization and repair. This remodeling process requires a complex turnover of the bone extracellular matrix, which is mediated in part by MMPs and TIMPs^36^. As they participate in almost all phases of bone repair, MMPs are active signaling molecules throughout the repair process^35^.

Thus, this increase in the expression of TIMP-1 in the first 24 hours after the implantation of biomaterial may be a stimulus to help the process of regulating the expression of MMP-9. Furthermore, this increase in TIMP-1 on the first day may be due to a stimulus to osteochondrogenic differentiation of adult mesenchymal progenitor cells^37^, as mesenchymal stem cells secrete high levels of TIMP-1 during differentiation into osteoblasts^38^,^39^.

## Conclusions

Given the above, it is essential to highlight the need to explore the study of the initial phases of a bone repair process when in contact with a biomaterial of interest, aiming to point out which biological mechanisms are involved and guarantee its biocompatibility in an adequate tissue-biomaterial interaction. In this sense, this study found interesting correlations with cytokine levels and gene expression, encompassing the local and systemic study. Therefore, it is possible to mention that the association HA/PLGA/BLEED has clinical potential, as it can act as a biocompatible biomaterial modulating the activity of some genes involved in the bone repair process, in addition to not causing exacerbated systemic inflammation.

## Data Availability

The data will be available upon request.
